# Generation of Gene-Knockout Mongolian Gerbils via CRISPR/Cas9 System

**DOI:** 10.3389/fbioe.2020.00780

**Published:** 2020-07-08

**Authors:** Yan Wang, Peikun Zhao, Zidai Song, Xiaoyan Du, Xueyun Huo, Jing Lu, Xin Liu, Jianyi Lv, Changlong Li, Meng Guo, Zhenwen Chen

**Affiliations:** Beijing Key Laboratory of Cancer Invasion and Metastasis Research, School of Basic Medical Science, Capital Medical University, Beijing, China

**Keywords:** Mongolian gerbils, CRISPR/Cas9, gene knockout, Cystatin C, Apolipoprotein A-II

## Abstract

The Mongolian gerbil (*Meriones unguiculatus*), a well-known “multifunctional” experimental animal, plays a crucial role in the research of hearing, cerebrovascular diseases and *Helicobacter pylori* infection. Although the whole-genome sequencing of Mongolian gerbils has been recently completed, lack of valid gene-editing systems for gerbils largely limited the further usage of Mongolian gerbils in biomedical research. Here, efficient targeted mutagenesis in Mongolian gerbils was successfully conducted by pronuclear injection with Cas9 protein and single-guide RNAs (sgRNAs) targeting Cystatin C (*Cst3*) or Apolipoprotein A-II (*Apoa2*). We found that 22 h after human chorionic gonadotropin (hCG) injection, zygote microinjection was conducted, and the injected zygotes were transferred into the pseudopregnant gerbils, which were induced by injecting equine chorionic gonadotropin (eCG) and hCG at a 70 h interval and being caged with ligated male gerbils. We successfully obtained *Cst3* and *Apoa2* gene knockout gerbils with the knockout efficiencies of 55 and 30.9%, respectively. No off-target effects were detected in all knockout gerbils and the mutations can be germline-transmitted. The absence of CST3 protein was observed in the tissues of homozygous *Cst3* knockout (*Cst3*-KO) gerbils. Interestingly, we found that disruption of the *Cst3* gene led to more severe brain damage and neurological deficits after unilateral carotid artery ligation, thereby indicating that the gene modifications happened at both genetic and functional levels. In conclusion, we successfully generated a CRISPR/Cas9 system based genome editing platform for Mongolian gerbils, which provided a foundation for obtaining other genetically modified gerbil models for biomedical research.

## Introduction

Mongolian gerbils (*Meriones unguiculatus*), belonging to the *muridae* family of *rodentia*, originated in the steppes of Mongolia and have been used as laboratory animals for about 80 years. They are beneficial for modeling various human diseases due to their unique features in cerebral vascular development, metabolism, pathogeny, epilepsy and auditory etcetera (Lay, 1972; Zhu et al., 2007). For instance, Mongolian gerbils are widely used for studying circle of Willis (CoW) variations and cerebral ischemia, as their types and incidence of CoW variations are similar to humans, and their single carotid artery ligation induced-stroke models are more effective and reproducible than those in other animals (Du et al., 2011, 2018; Martinez et al., 2012). A hereditable spontaneous diabetic gerbil line established by us previously presented moderate hyperglycemia, hyperinsulinemia, obesity and diabetic pathophysiological lesions revealing gerbil’s value in studying diabetic pathogenesis (Boquist, 1972; Li et al., 2016). What’s more, the susceptibility and disease progression of *Helicobacter pylori* (*H. pylori*) in gerbils is highly comparable to that in humans making gerbils advantageous for studying *H. pylori*-related gastropathy (Rieder et al., 2005; Wei et al., 2010; Noto et al., 2016). Recently, the whole-genome sequencing of Mongolian gerbils and *Psammomys obesus* (*P. obesus*) has been completed (Hargreaves et al., 2017; Zorio et al., 2018), which provided a referential database for future genome editing programs of such organism. However, no gene-editing Mongolian gerbil models have been reported before, which limited the further usage of Mongolian gerbils in biomedical research. Therefore, it is essential to establish an effective genome editing platform in Mongolian gerbils.

The clustered regularly interspaced short palindromic repeats (CRISPR)/CRISPR-associated protein 9 (Cas9) is a revolutionary gene editing tool (Ma et al., 2014). By employing a single guide RNA (sgRNA) chimera consisting of a fusion between crRNA and tracrRNA and Cas9 protein, it generates targeted DNA double-strand breaks (DSBs) and nonhomologous end joining (NHEJ)-induced imperfect repair, which accounts for unintended nucleotide insertions/deletions (indels) and subsequent gene knockout (Jinek et al., 2012; Jiang and Doudna, 2017; Shen et al., 2017). CRISPR/Cas9 has prominent versatility, efficiency, simplicity and sequence-specificity over other gene editing tools, and has been widely used in many organisms (Ruan et al., 2017), suggesting that CRISPR/Cas9 system can be a valid tool for generating gene knockout Mongolian gerbils.

Cystatin C (CST3), as the most important secreted cysteine inhibitor, is extensively distributed in human organs and body fluids, and functions in a variety of physiological processes, such as proenzyme degradation and regulation (Seronie-Vivien et al., 2008). A series of clinical investigations and *in vitro* studies also reveal its role in tumorigenesis, cardiovascular and kidney diseases (Shi et al., 1999; Odutayo and Cherney, 2012; Leto et al., 2018). Recently, CST3 has also emerged as a potential neuron protector in neurodegenerative diseases like Alzheimer’s disease (AD), Parkinson’s disease (PD), and amyotrophic lateral sclerosis (ALS) (Mathews and Levy, 2016; Zou et al., 2017; Watanabe et al., 2018). In our inbred strain of ischemia-prone Mongolian gerbils, *Cst3* was identified as one of the differential expression (DE) genes, which may link with different types of CoW (Li et al., 2015). We also found that CST3 was involved in vascular development by modulating endothelial cell proliferation and migration (Li et al., 2018). However, the roles of CST3 in many physiological and pathological processes, especially the roles in brain protection, have not been fully clarified by using gene-edited animal models.

Mongolian gerbils have the tendency to develop diabetes (Boquist, 1972; Vincent et al., 1979). Recently, we have established a spontaneous diabetic gerbil inbred strain after a 10-year selective breeding (Li et al., 2016). By using our diabetic models, *Apolipoprotein A-II* (*Apoa2*) was identified as a DE gene in skeletal muscle (Guo et al., 2020). APOA2 is the second most common component of high-density lipoproteins (HDL), stabilizes HDL by suppressing their remodeling by lipases (Warden et al., 1993). The polymorphism of *Apoa2* is related to lipid metabolism, obesity and atherosclerosis in human (Zaki et al., 2014; Lai et al., 2018). Low-density lipoprotein (LDL) is the main cholesterol carrier in both human and gerbils, whereas HDL is the major lipoprotein and functional cholesteryl ester transfer protein (CETP) is absent in mice (Maiga et al., 2014), revealing that Mongolian gerbils may be a proper model to study functions of lipoprotein, such as APOA2. However, the precise role of APOA2 in metabolism, especially in gerbil metabolism, has not been clarified.

In the present study, we reported the first successful strategy for CRIPSR/Cas9-mediated gene editing in Mongolian gerbils, and produced Cystatin C (*Cst3*) knockout and Apolipoprotein A-II (*Apoa2*) knockout gerbils with high efficiency.

## Materials and Methods

### Animals and Ethics

All experimental and animal program management in this study was consistent with the guidelines of the Capital Medical University Animal Experiments and the Experimental Animals Management Committee and the Animal Research: Reporting of *in vivo* Experiments guidelines (Kilkenny et al., 2010). The study protocol was approved by the Animal Experimental and Experimental Animal Ethics Committee of the Capital Medical University (AEEI-2017-032). The closed colony and the ischemia-prone inbred Mongolian gerbils used in this study were domesticated and cultivated in laboratory animal facilities of Capital Medical University with a humidity of 40–65%, a temperature of 22 ± 4°C and a 12L: 12D light cycle.

### Preparation of sgRNAs

sgRNAs targeting different genes were designed according to the Feng Zhang’s online protocol^1^. A specific sequence complementary to the sticky end of BsaI was added to the sgRNA sequences, and the Oligo DNA synthesis primers were obtained by chemical synthesis (Supplementary Table S1). A pair of oligonucleotides for each sgRNA was annealed and cloned into BsaI sites of pX330 expression vector (Addgene plasmid ID: 42230). To obtain transcription templates, sgRNA sequences were amplified from sgRNA-pX330 expression vector by using specific primers (Supplementary Table S2). After sequencing, sgRNAs were transcribed by using mMESSAGE mMACHINE^®^ T7 ULTRA Transcription Kit (Invitrogen, AM1345, United States) and purified by MEGAclear^TM^ Transcription Clean-Up Kit (Invitrogen, AM1908). The purity of sgRNAs was confirmed by RNA concentration measurement and RNA electrophoresis.

### Superovulation and Preparation of Foster Mothers

According to our previous reports (Tang et al., 2015), 6–8-week-old female closed colony Mongolian gerbils were superovulated by intraperitoneal injection of 10 IU equine chorionic gonadotropin (eCG) (Ningbo Second Hormone Factory, China) at 4 pm to 5 pm on the first day and followed by injection of 10 IU human chorionic gonadotropin (hCG) (Ningbo Second Hormone Factory) at 2 pm to 3 pm on the fourth day, and then were mated with fertile males. Embryos from oviducts were collected at 19 h after caging. Ten-week-old female closed colony gerbils were mated with ligated males to produce pseudopregnant foster mothers. What’s more, trays were used here to examine the copulation plugs after mating in gerbils.

### Microinjection and Embyro Transfer

For cytoplasmic injection, sgRNA (50 ng/μL) and Cas9 protein (32 ng/μL) (NEB, M0386T, United States) was diluted and mixed by ddH_2_O without ribozyme. 3–7 pL solutions were injected into an embryo cytoplasm. The injected embryos were cultured in M2 medium (Sigma-Aldrich, M7167, United States) at 37°C in 95% humidified air and 5% CO_2_ over 0.5 h. The embryos with normal morphology were transferred into the oviduct of a pseudopregnant Mongolian gerbil. The foster mothers naturally delivered and raised their pups.

### Analysis of Offspring Genotypes

DNA was extracted from pup’s ears by a phenol chloroform extracting method. PCR was performed by using DreamTaq^TM^ Hot Start Green PCR Master Mix (Thermo Fisher Scientific, K9021, United States) in accordance with the following conditions: pre-denaturation at 95°C for 5 min; 35 cycles of denaturation at 95°C for 30 s, annealing at 60°C for 30 s and extension at 72°C for 1 min; 72°C for 7 min with gene specific primers (Supplementary Table S3). PCR products were analyzed by Sanger sequencing. The PCR products which harbor mutations were subcloned into a pMD19-T vector using pMD19-T Vector Clone Kit (Takara, 6013, Japan). Each subcloned vector was analyzed by direct sequencing.

### Preparation of the Anti-Mongolian Gerbil CST3 Polyclonal Antibody

Total RNA was extracted from Mongolian gerbil brain and reversely transcribed into cDNA. The coding domain sequence (CDS) region of Mongolian gerbil *Cst3* was amplified by forward primer “5′-ATGGCTAGCCCACTACGATCC-3′” and reverse primer “5′-TTAAGCGCTTTTGCAGCTGGA-3′”. The DNA was ligated to the pMD19-T vector for TA cloning and sequencing. The sequence of *Cst3* CDS region was subjected to codon optimization, *in vitro* synthesis, enzyme digestion and vector ligation, and then the *in vitro* expression vector of SUMO-CST3 was obtained. The recombinant SUMO-CST3 protein was induced to express with 1 mmol/L isopropyl β-D-Thiogalactoside (IPTG) at 37°C for 12 h. Then the protein was purified following the inclusion body protein purification process and harvested at a concentration of 6.0 mg/mL. A rabbit was immunized with the purified recombinant CST3 protein four times to obtain an anti-CST3 polyclonal antibody.

### Western Blotting Analysis

Total protein was extracted using a tissue protein extraction kit (CWBio, CW0891M, China) containing protease inhibitors (PMSF) (CST, 8553S, United States) and quantified by Pierce^TM^ Rapid Gold BCA Protein Assay Kit (Thermo Fisher Scientific, A53225). Protein lysates were separated by 15% SDS-PAGE at 120 V and electrotransferred to 0.22 μm nitrocellulose blotting membranes (PALL, 66485, United States) at 70 V for 2 h. After blocking in 5% non-fat milk (BD, 232100, United States) for 1 h, the membranes were incubated with the anti-Mongolian gerbil CST3 polyclonal antibody or the anti-GAPDH antibody (CST, 5174, United States) overnight at 4°C. After washes and incubation in 1:5000 dissolved secondary antibodies for 1 h at room temperature, the membranes were visualized using Pierce^TM^ ECL Western Blotting Substrate (Thermo Fisher Scientific, 32106) and scanned by Gel DocXR System in Bio-Rad Laboratories (Bio-Rad, United States).

### Analysis of Off-Target Sites

To assess the site-specific cleavage in Cas9/sgRNA-mediated mutant gerbils, the potential off-target sites were searched in the whole Mongolian gerbil genome^2^ and were selected based on the following rules: (1) the sequences had no more than four mismatches to the sgRNAs, (2) the protospacer-adjacent motif (PAM) sequences were NGG or NAG. And we scored the potential off-target sites by using algorithms from CasFinder^3^ (Aach et al., 2014) (Supplementary Table S4). A higher score meant the sites had more chance to bind with Cas9–sgRNA complexes. Ten sites with the highest scores were amplified from all founders. The primers were listed in Supplementary Tables S5, S6. PCR was performed in the following conditions: pre-denaturation at 95°C for 5 min; 35 cycles of denaturation at 95°C for 30 s, annealing at 60°C for 30 s and extension at 72°C for 1 min; 72°C for 7 min. PCR products were analyzed by Sanger sequencing.

### Establishment of Cerebral Ischemia Animal Model and Assessment of Neurological Deficits

Ten to twelve, 12–16-week-old *Cst3* knockout (*Cst3*-KO) homozygous gerbils (half males and half females) and their wild type (WT) controls were anesthetized by diethyl ether, respectively. Unilateral common carotid artery ligation was performed on all animals.

After a 1 h ligation, the vertical grid experiment, which was improved based on the climbing board test and the vertical pole test (Yonemori et al., 1998; Bouet et al., 2007), was performed here to evaluate the gerbil’s forelimb strength, grasping ability, motion coordination and responsiveness. Place the gerbils on the iron wire with a 0.5 cm spacing horizontally. After balancing for 10 s, the iron net was turned quickly to be vertical, and whether the gerbil could fix its body on the wire net and whether it could crawl was observed. If the gerbil could not catch the iron net and fell off within 20 s, it was recorded as “3 points;” if it fell within 20–40 s, it was recorded as “2 points;” if the gerbil could keep itself on the iron net or could continuously crawl for more than 40 s, “0 point” was recorded.

The Zea-Longa method is widely used to measure neurological deficits in middle cerebral artery occlusion-induced cerebral ischemia models in mice and rats (Longa et al., 1989). However, the phenotypes observed in unilateral common carotid artery ligation-induced cerebral ischemia gerbils were progressively more severe with the prolongation of ischemia time, and the phenotypes were more variable, compared with those in mice and rats (Ito et al., 2013). Therefore, to describe the severity of the symptoms more accurately within 10 h after ligation in gerbils, we established a “10-point evaluation method.” If one of the following phenotypes (eyelid drooping, limb deflection, spin, spin radius less than 3 cm, jump or excavation, turnover, loss of consciousness, incontinence, death during 5–10 h, death within 5 h) occurs, we appended one point to the injury score, and the higher the score is the more severe the disease is.

### 2, 3, 5-Triphenyl-Tetrazolium Chloride Solution (TTC) Staining

TTC was conducted as reported previously (Chelluboina et al., 2014). TTC was conducted as reported previously. Briefly, after 10 h of ligation and euthanasia by cervical dislocation, an intact brain was quickly isolated, and frozen in −20°C for 20 min, and then cut into 2 mm thick brain slices along the coronal position with blades. Next, the brain slices were placed into a 2% TTC solution (Solarbio, T8170), stained for 1 h at room temperature, and fixed overnight by 4% paraformaldehyde at 4°C. The ipsilateral brain slices were captured and analyzed by Image J to calculate the infarct volumes.

### Identification of the Anatomical Patterns of CoW in Gerbils

The anatomical structures of the posterior communication artery (PCoA) and the anterior communication artery (ACA) in WT and *Cst3*-KO gerbils were observed under a stereoscope after euthanasia and autopsy. According to the previous reports (Du et al., 2006), all gerbils used here were identified absent of PCoA. And the types of ACA were classified as complete, incomplete (the left, the right or the bilateral ACAs were much smaller) and absent (the left, the right or the bilateral ACAs were absent).

### Metabolic Phenotyping of *Apoa2*-KO Gerbils

Metabolic phenotyping of 12-week old *Apoa2*-KO gerbils were analyzed. After 16 h of fasting, body weight and serum glucose levels of *Apoa2*-KO gerbils were measured. After 16 h of fasting, the animals were given D-glucose orally at 2 g/kg body weight to test oral glucose tolerance test (OGTT). The blood glucose values were measured using a glucometer (SANNUO, China).

### Statistical Analysis

All data were expressed as “mean ± SEM.” The differences of CoW patterns between WT and *Cst3*-KO gerbils were analyzed by the chi-squared test, and other data were analyzed by *t*-test or the variance analysis with SPSS 21.0 software. *p <* 0.05 denotes statistically significant. All experiments were repeated at least 3 times.

## Results

### Embryo Transfer of Mongolian Gerbils

**FIGURE 1:**
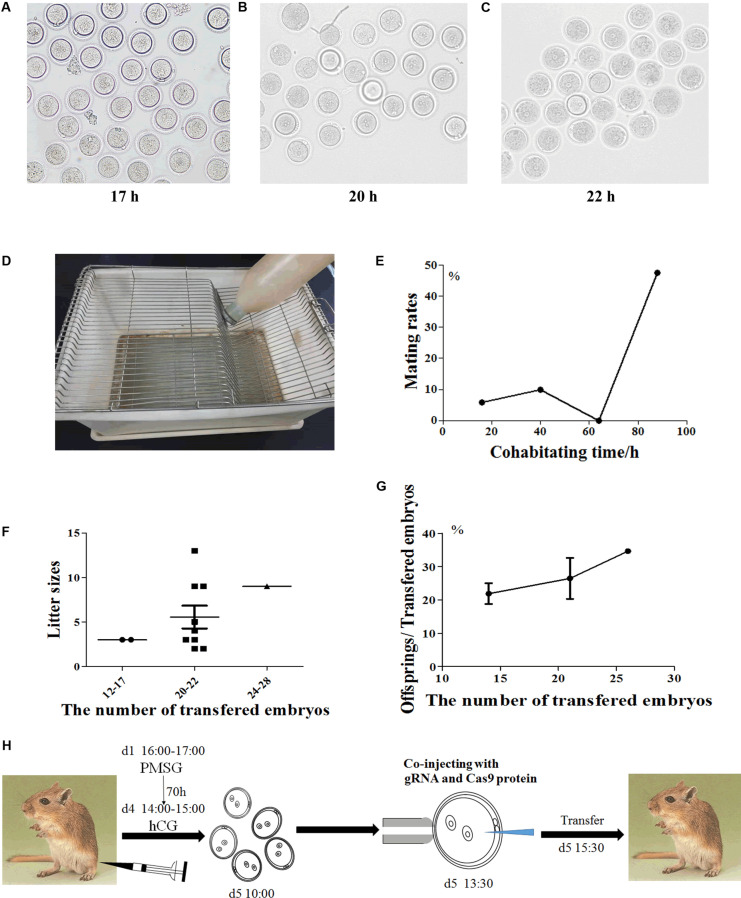
The procedure of embryo manipulation in generating CRISPR/Cas9-induced gene knockout gerbils. The morphology of the fertilized eggs of gerbils after hCG injection 17 h **(A)**, 20 h **(B)** and 22 h **(C)**, respectively. The tray for detecting gerbil mating vaginal plus **(D)**. The mating rates after natural cages at different timepoints **(E)**. The numbers of progeny after different numbers of fertilized eggs were transferred into recipient gerbils **(F)**. The numbers of live offspring/the numbers of transferred fertilized eggs **(G)**. The optimized experimental operation flow chart **(H)**.

The reproductive characteristics of Mongolian gerbils, such as the time of sexual maturity and the duration of estrous cycle and gestation, are greatly different from those of mice (Nishino and Totsukawa, 1996; Chen et al., 2014; Gonzalez, 2016; Yoshida et al., 2016; Vidal and Filgo, 2017). And resulting from monogamy and strong aggressiveness, death often occurs when heterosexual gerbils mate. Thus, it is challenging for embryo manipulation of Mongolian gerbils. To establish the CRISPR/Cas9 system in gerbils, we first developed the procedure of embryo manipulation in gerbils. We previously proved that the best superovulation protocol was to inject 10 IU eCG and hCG at a 70 h interval (Tang et al., 2015). Here, the timepoint of microinjection was assessed by identifying the appearance of pronucleus. 17 h after hCG injection, the pronucleus of zygotes began to appear, the proportion of zygotes with pronucleus increased to 80% at 20 h after hCG injection, and the proportion peaked (about 90%) at 22 h after hCG injection (Figures 1A–C). Thus, the optimal timepoint for microinjection in Mongolian gerbils was set at 22 h after hCG injection.

Next, we optimized the strategy for preparing foster mothers. Given that gerbils’ copulation plugs fall off easily, trays were used to examine the vaginal plugs of gerbils (Figure 1D). After mating with a male, the vaginal plug number of each female ranged from 1 to 5 (was usually 3–4) per night (data not shown). Notably, the natural mating rate was less than 10% within the first 3 days (0–64 h), but this rate increased to 47.62% on the fourth day of cohabitation (88 h) (Figure 1E and Table 1). Meanwhile, the mating rate of gerbils treated with 10 IU eCG and hCG was 57.69%, while 5 IU or 7.5 IU eCG/hCG injection only caused 16.67% (1/6) and 0 (0/3) couples to mate, respectively (Table 1). No significant differences were found between the pup numbers of the foster mothers with natural estrus and with hormone-induced estrus (data not shown). And litter sizes increased with the increasing number of transferred embryos. When 20–22 embryos were transferred, the offspring number was about 5, close to that of naturally-mating gerbils (Figures 1F,G).

**TABLE 1:** The comparison between hormone-induced and natural mating rates in gerbils.

	eCG doses	hCG doses	Cohabitation time	Mating rates
Hormone-induced mating	5 IU	5 IU	16 h	16.67% (1/6)
	7.5 IU	10 IU	16 h	0 (0/3)
	10 IU	10 IU	16 h	57.69% (15/26)
Natural mating			16 h	5.88% (1/17)
			40 h	10% (1/10)
			64 h	0 (0/9)
			88 h	47.62% (10/21)

Taken together, the optimal procedure of superovulation and embryo transfer for generating CRISPR/Cas9-induced gene knockout gerbils was as follows: 6-week-old female gerbils were injected with10 IU eCG/hCG at a 70-h interval, then were caged with males. Zygotes were collected at 17 h after hCG injection and microinjection was performed at 22 h after hCG. To prepare pseudopregnancy gerbils, 10–12 week-old female gerbils were caged with ligated males after injection with 10 IU eCG/hCG at a 70 h interval. The vaginal plug was checked by a tray, and 20–22 fertilized eggs were transferred into a unilateral fallopian tube of a foster mother (Figure 1H).

### Generation of Cystatin C Knockout (*Cst3*-KO) and Apolipoprotein A-II Knockout (*Apoa2-*KO) Gerbils by Cytoplasmic Microinjection of sgRNA and Cas9 Protein

**FIGURE 2:**
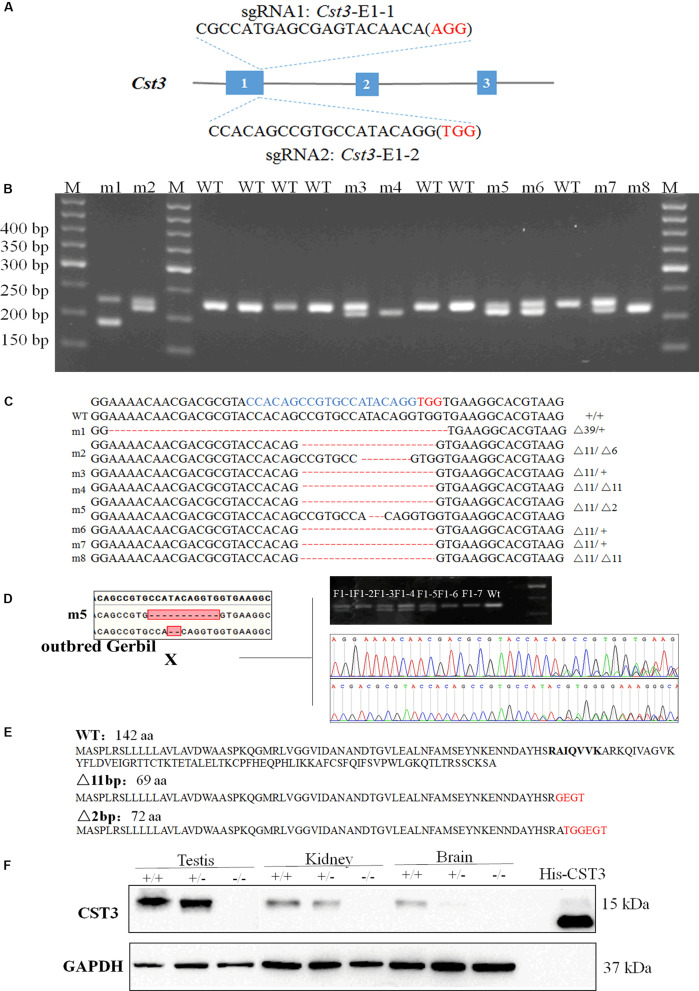
Genotypic identification of *Cst3*-KO gerbils. The sgRNA sequences targeted to exon 1 of *Cst3* **(A)**. PCR amplification and agarose gel electrophoresis of *Cst3* gene **(B)**, and their genotypes **(C)** of gerbil founders. The target sequences were highlighted in blue and the PAM sequences were highlighted in red. The founders were mated with the ischemia-prone inbred gerbils, and the mutations were inherited by the F1 generation **(D)**. CST3 amino acid sequences in wild type (WT) Mongolian gerbils and the predicted amino acid sequences in *Cst3* mutant gerbils **(E)**. Western blotting was employed to measure the expression of CST3 protein in *Cst*-KO gerbils, and the purified recombinant CST3 protein with His tag was treated as a positive control **(F)**.

**FIGURE 3:**
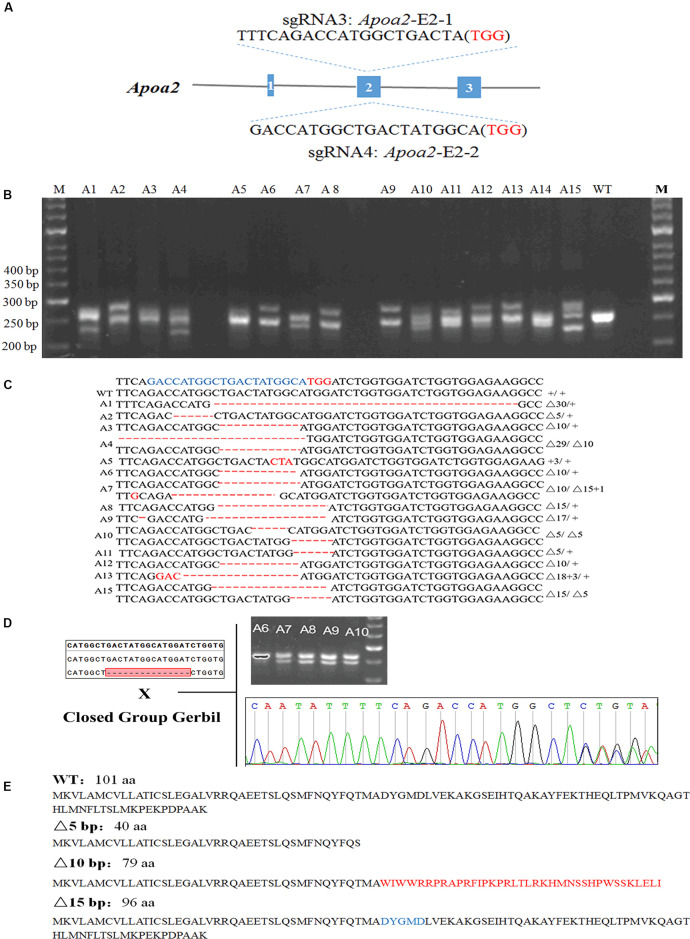
Genotypic identification of *Apoa2*-KO gerbils. The sgRNA sequences targeted to exon 2 of *Apoa2* **(A)**. PCR amplification and agarose gel electrophoresis of *Apoa2* gene **(B)** and genotypes **(C)** of a part of gerbil founders. The target sequences were highlighted in blue and the PAM sequences were highlighted in red. The founders were mated with closed group gerbils, and the mutations were inherited by the F1 generation **(D)**. APOA2 amino acid sequences in WT gerbils and the predicted amino acid sequences in *Apoa2* mutant gerbils **(E)**.

In order to construct gene knockout Mongolian gerbils, we designed sgRNAs targeting gerbil *Cst3* and *Apoa2* gene, because of their essential roles in multiple physiological and pathological processes (Basiri et al., 2015; Mathews and Levy, 2016; Lai et al., 2018; Leto et al., 2018). Two sgRNAs pairs targeting *Cst3* or *Apoa2* in gerbils were designed by Feng Zhang’s online protocol, respectively (Figures 2A, 3A). 50 ng/μL sgRNA and 32 ng/μL Cas9 protein was microinjected into the cytoplasm of fertilized eggs of Mongolian gerbils. As shown in Table 2, the survival rates of the injected zygotes were 81.3% (80.5–88.1%), which were comparable to those in mice and hamsters (Fan et al., 2014; Harms et al., 2014). The gene editing efficiencies in *Cst3*-KO and *Apoa2*-KO gerbils were 55% (11/20) and 30.9% (17/55), respectively, similar with those in mice (50%), rats (29–53%) and hamsters (14.3–88.9%) (Fan et al., 2014; Guan et al., 2014; Harms et al., 2014).

**TABLE 2:** The generation of the two knockout gerbils.

sgRNA	sgRNA concentrations	Cas9 concentrations	Injected embryos	Survival embryos (% of injected embryos)	Transferred embryos	Live pups (% of transferred embryos)	KO (% of live pups)
*Cst3*-E1-1	50 ng/μL	32 ng/μL	106	85 (80.2)	85	12 (14.1)	0 (0)
*Cst3*-E1-2			155	127 (89.1)	127	20 (15.7)	11 (55)
*Apoa2*-E2-1			42	37 (88.1)	37	5 (13.5)	0 (0)
*Apoa2*-E2-2			308	248 (80.5)	248	55 (22.2)	17 (30.9)
							

*Cst3* gene of 11/20 pups was mutant after injection of Cas9 and sgRNA *Cst3*-E1-2 (Figure 2A). Sanger sequencing showed that Cas9/*Cst3*-E1-2 sgRNA targeted exon 1 of *Cst3*, and resulted in deletions of 2, 6, 11, or 39 nucleotides (fetuses with a 6-bp or 39-bp deletion died before birth) (Figures 2B,C and Supplementary Figure S2). All the *Cst3* mutations were transmitted to offspring by mating with the ischemia-prone inbred gerbils (Figure 2D). The 2 bp or 11 bp deletion of *Cst3* was predicted to cause the truncation of the protein, which can reduce its length to 72 aa or 69 aa, respectively (Figure 2E). To confirm the absence of CST3 at protein levels in the *Cst3-*mutant gerbils, a rabbit anti-Mongolian gerbil CST3 polyclonal antibody was produced first, and a single band at the predicted molecular weight revealed its specificity (Supplementary Figures S3A,B). Western blotting showed that CST3 expression decreased in the heterozygous gerbils and was completely absent in *Cst3*-KO homozygous gerbils (Figure 2F).

17/55 pups were genetically modified after injection of Cas9/sgRNA *Apoa2*-E2-2 (Figure 3A). Sanger sequencing showed that Cas9/*Apoa2*-E2-2 sgRNA targeted exon 2 of *Apoa2*, and resulted in deletions of 3, 5, 10, 15, 29, or 30 nucleotides (Figure 3B and Supplementary Figure S4). Their genotypes were summarized in Figure 3C (fetuses with a 5-bp, 10-bp or 30-bp deletion died before birth). All *Apoa2* mutations can be germline-transmitted by mating with our closed group gerbils (Figure 3D). The amino acid sequences expressed by different *Apoa2* mutations were predicted. Except for the 15 or 30 bp deletion, all other genotypes exhibited premature termination and produced truncated proteins (Figure 3E). In summary, we successfully generated a CRISPR/Cas9 protocol for genome editing in Mongolian gerbils firstly.

### Off-Target Analysis

CRISPR/Cas9 system may introduce off-target effects (Fu et al., 2013; Wu et al., 2014). Because the Mongolian gerbil genome was not added into the database of the off-target analysis websites such as CasFinder. Here, we first developed algorithms referring to CasFinder, and by using the algorithms we screened the Mongolian gerbil genome. The sequences containing ≤ 4 bp mismatches were considered as potential off-target sites, and the indel events that occurred within 20 bp up-stream or down-stream of the potential off-target sequences were considered as off-target effects. Ten off-target sites with the highest scores were amplified. PCR genotyping and Sanger sequencing showed that no off-target effects were observed in the founders of both *Cst3*-KO *and Apoa2-*KO gerbils (Supplementary Figures S5, S6).

### Aggravated Brain Damage in Cerebral Ischemic Gerbils of *Cst3*-KO Gerbils

**FIGURE 4:**
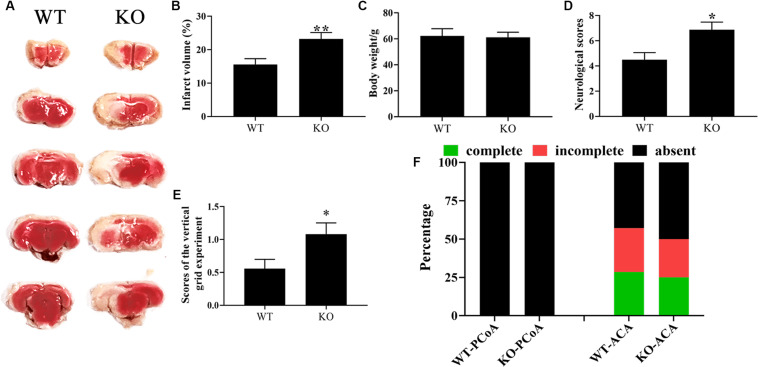
The deficiency of CST3 aggravated brain damage in cerebral ischemic gerbils. Brain infarct volumes were evaluated by TTC staining at 10 h after unilateral carotid artery ligation in gerbils, *n* = 10–12 per group **(A)**. And the proportions of infarct volume were calculated by image J **(B)**. The body weight of the *Cst3*-KO group and the WT group **(C)**. The neurological deficit scores were assessed at 10 h after unilateral carotid artery ligation **(D)**. The scores of the vertical grid experiment, which was performed to evaluate the gerbil’s forelimb strength, grasping ability, motion coordination and responsiveness, were measured at 1 h after unilateral carotid artery ligation **(E)**. The percentages of each type of PCoA and ACA in WT and *Cst3*-KO gerbils **(F)**, *n* = 20–21 per group. * and ** denote *p* < 0.05 and *p* < 0.01, respectively.

CST3 has emerged as a potential neuroprotective and angiogenesis function in neurodegenerative disease like AD, PD, and ALS (Mathews and Levy, 2016; Zou et al., 2017; Watanabe et al., 2018). The variation of CST3 expression level may link with different types of CoW in our inbred strain of ischemia-prone Mongolian gerbils (Li et al., 2015; Du et al., 2018). However, whether CST3 is involved in CoW development and brain recovery after cerebral ischemia are not fully understood. Here, to verify the functional deficiency of CST3 in *Cst3*-KO gerbils and to assess the role of CST3 in stroke, we ligated unilateral carotid arteries of gerbils, and found that the brain infarct areas of *Cst3*-KO gerbils (23.2 ± 1.91%) were significantly larger than those of WT gerbils (15.6 ± 1.75%) by the TTC staining assay (Figures 4A,B). CST3 deficiency also aggravated the neurological function deficits and severely impaired the grip strength of forelimbs (Figures 4D,E). On the other hand, *Cst3* knockout did not influence body weight and the anatomical patterns of PCoA and ACA in gerbils (Figures 4C,F). Therefore, our data indicated that CST3 was functional deficient in *Cst3*-KO gerbils and CST3 has brain protective effects on cerebral ischemia.

## Discussion

The whole-genome sequencing of Mongolian gerbils has been recently completed (Zorio et al., 2018). However, no gene editing of gerbils has been ever been reported. In the current study, by optimizing the experimental procedures for microinjection, fertilized egg transfer, and recipient preparation, we successfully obtained germline-transmitted *Cst3*-KO and *Apoa2*-KO gerbils using a CRISPR/Cas9 system, and no off-target effects were detected in all knockout founders. Interesting, to further assess the functional deficiency of CST3 in *Cst3*-KO gerbils and to determine the role of CST3 in stroke, the unilateral carotid arteries of *Cst3*-KO gerbils were ligated and the animals showed more severe brain damage and neurological deficits than WT controls, indicating the validation of our knockout system in gerbils and a neuroprotective role of CST3 in cerebral ischemia.

The reproductive physiology and behavior of Mongolian gerbils is greatly different from that of mice and rats. Mouse sexual maturity occurs at 6–8 weeks old, estrous cycle lasts for 4–5 days, and gestation period is 19–21 days (Gonzalez, 2016; Yoshida et al., 2016; Vidal and Filgo, 2017). In contrast, gerbils don’t attain sexual maturity until 10–12 weeks old, their sexual cycle lasts for 4–6 days, and gestational period is 25–27 days (Nishino and Totsukawa, 1996; Chen et al., 2014). Consistently, we previously reported that the superovulation protocol for gerbils was to inject 10 IU eCG and hCG at a 70 h interval (Tang et al., 2015), longer than that in mice. To avoid the formation of chimeric animals, it is important to measure the time point that pronucleus occurs (Sato et al., 2018). Mouse pronucleus is usually observed at 15–20 h after hCG injection, while our study showed that pronucleus formation in gerbils was at 17–22 h after hCG injection.

The preparation of receipt gerbils and embryo transfer is another challenge. In gerbils, the mating time is uncertain and the mating rates within the first 3 days was less than 20% after natural cages. Norris and Adams (1981) reported that the copulation plug in gerbils does not disappear until the next morning. But in our gerbil line, vaginal plugs in 29/30 gerbils cannot be detected even at 4 am in the next morning after cohabitation. Thus, the trays were used to collect the dropped vaginal plugs, which indicates successful mating. It is noticeable that the mortality of gerbils after natural cages was about 10–20%, due to their monogamy and strong aggressiveness. A previous study suggests that the conception rates of gerbils increased by hormone injection (Wu, 1974). Here we induced estrus in recipient females by hormone injection, which significantly increased the mating rates and decreased the mortality in gerbils. In our study, the progeny numbers were 5–7 after transferring 20–22 eggs into a receipt gerbil, which was a suitable embryo transfer approach concluding from its similar litter size to that after natural mating (Zhu et al., 2007). Therefore, the optimized procedures of embryo manipulation provided a basis for CRISPR/Cas9 gene editing in Mongolian gerbils.

Here we generated gene knockout gerbils by microinjection of Cas9 proteins and sgRNAs into the cytoplasm of fertilized eggs of Mongolian gerbils, for Cas9 protein injection can mitigate off-target effects to some extent, relative to Cas9 mRNA injection. In our study, the survival rates of the microinjected eggs were greater than 80%, indicating that the damage of microinjection is negligible in gerbils. Our gene editing efficiencies in *Cst3*-KO and *Apoa2*-KO gerbils were 55 and 30.9%, respectively, similar with those in mice (50%), rats (29–53%), golden Syrian hamsters (14.3–88.9%), monkeys (46.47%), and zebrafish (24.1–59.4%) (Hwang et al., 2013; Fan et al., 2014; Ni et al., 2014; Chen et al., 2015; Bakondi et al., 2016; Vejnar et al., 2016; Ryu et al., 2019). Furthermore, the mutations can be germline transmitted. CST3 was absent in *Cst3*-KO homozygous gerbils at protein levels. Taken together, we successfully constructed *Cst3*-KO and *Apoa2-*KO gerbils with different genotype using CRISPR/Cas9 technology.

CRISPR/Cas9 system can lead to off-target mutations due to the effect of mismatch tolerance in a sequence and position dependent manner (Fu et al., 2013; Hsu et al., 2013). In mice, off-target effects only occur in the sites that have one or two base mismatches depending on their positions at the sgRNAs, and do not occur in the sites with ≥ 3-base pair mismatches (Yang et al., 2013). Therefore, by optimizing sgRNA design, CRISPR/Cas9-induced off-targeting events *in vivo* are very rare (Dong et al., 2019). Few off-target effects are discovered in CRIPSR/Cas9-mediated gene-modified pig, monkey, *Drosophila*, golden Syrian hamster, and goats (Hwang et al., 2013; Fan et al., 2014; Guan et al., 2014; Ni et al., 2014; Vejnar et al., 2016). Consistently, in the present study, no off-target effects were detected in the *Cst3*-KO or *Apoa2-*KO gerbils. The possible reason is that all predicted off-sites in our study had two or more base pair mismatches, which dramatically reduced non-specific Cas9 cleavage. Moreover, whether the sgRNA/Cas9-independent genomic mutations occurred is still unclear, which needs further investigations by employing the genome-wide, unbiased method, such as genome-wide, unbiased identification of DSBs enabled by sequencing (Guide-seq) and high-throughput genome wide translocation sequencing (HTGTS) (Tsai et al., 2015; Yin et al., 2018).

*Cst3*-KO mice are healthy and fertile, and grow at a normal rate (Huh et al., 1999). Consistently, we had not found any differences in body weight, development and fertility between *Cst3*-KO and wild type gerbils. Recently, a protective role of CST3 against neuronal damage is emerging. For instance, CST3 increases neuron viability by inhibiting autophagy and cathepsin B (Cat B) in Cu/Zn-superoxide dismutase (SOD1)-mutant, or cytotoxicity-exposed neuroblastoma cell lines and primary cultured motor neuronal cells (Tizon et al., 2010; Watanabe et al., 2014). CST3 administration also promotes neuronal survival and angiogenesis by increasing VEGF in PD neurovascular units (Zou et al., 2017). CST3 maintains blood-brain barrier integrity by regulating caveolin-1 expression after stroke in mice. And CST3 deletion aggravated brain damage after ischemia-reperfusion in mice (Olsson et al., 2004). Here, to further analyze the phenotypes and to verify the functional deficiency in *Cst3-*KO gerbils, we ligated unilateral carotid arteries of gerbils. The *Cst3*-KO gerbils displayed more cerebral ischemic areas and higher neurological damage scores than did wild-type gerbils, indicating that CST3 was involved in post-ischemic brain protection. Consistently, the inhibitor treatment of Cat B and L, suppressing targets of CST3 (Melander et al., 2009; Lopes et al., 2019), also reduces infarct volumes and improves neurological deficits in cerebral ischemic rats (Xu et al., 2014). In addition, both WT and *Cst3*-KO gerbils showed the absence of PCoA, and no differences in the types of ACA, revealing that CST3 null did not change the anatomy structures of PCoA and ACA in gerbils. Therefore, our data indicated that CST3 alleviated post-ischemic brain damage. By testing a series of behavioral experiments, we also found that the aging *Cst3*-KO gerbils showed a decrease of social discovery and depression trend (data not shown), indicating that CST3 may have multiple protective roles in brain.

In the present study, *Apoa2*-KO gerbils had no significant differences in body weight, blood glucose and glucose tolerance, compared with controls (Supplementary Figures S7A–C). In human, increasing APOA2 synthesis does not influence glucose tolerance (Kalopissis et al., 2003). *Apoa2* variants does not link with type 2 diabetes susceptibility (Duesing et al., 2009). However, *Apoa2*-KO mice display decreased blood glucose, HDL, cholesterol and free fatty acid (FFA) (Weng and Breslow, 1996). *Apoa2* overexpressed mice display increased body weight, blood glucose, HDL, cholesterol and FFA (Castellani et al., 2001), revealing the inconsistency of APOA2 functions in gerbils and mice. APOA2 also play different roles in human and mice atherosclerosis. In human, increasing APOA2 synthesis decreases the incidence of atherosclerosis (Kalopissis et al., 2003). Whereas, *Apoa2* overexpressed mice develop atherosclerotic lesions even on a chow diet (Boisfer et al., 2002). The explanation of the differences is probable that HDL is mice’s major lipoprotein, in contrast low-density lipoprotein (LDL) is the main cholesterol carrier in human and gerbils (Hegsted and Gallagher, 1967; Maiga et al., 2014). And the particle size and antioxidant properties of HDL and effects of high fat/high cholesterol diet in mice are different from those in gerbils and human (Difrancesco et al., 1990; Kalopissis et al., 2003). Taken together, gerbils may be a preferable model to study lipid metabolism. And the high-fat diet treatment and more detailed investigations in *Apoa2*-KO gerbils are required in the future study.

In conclusion, we successfully constructed *Cst3*-KO and *Apoa2*-KO Mongolian gerbils with efficient germline transmission by using CRISPR/Cas9 technology. The CRISPR/Cas9 system in gerbils provides a powerful tool for researching biological characteristics of gerbils, expands the use of gerbils as a model organism, and benefits for comparative biological studies of rodents. Furthermore, due to the alternative advantages of gerbils for modeling various human diseases (Lay, 1972; Zhu et al., 2007), the gene-editing system also builds a basis for researching human diseases using gerbil models.

## Data Availability Statement

The original contributions presented in the study are included in the article/Supplementary Material, further inquiries can be directed to the corresponding authors.

## Ethics Statement

The animal study was reviewed and approved by the Animal Experimental and Experimental Animal Ethics Committee of the Capital Medical University (AEEI-2017-032).

## Author Contributions

YW: methodology, data curation, and writing – original draft preparation. PZ: software. ZS: validation. XD: visualization. JLu: supervision. XL: investigation. JLv: software. MG, CL, and ZC: experimental design. XH, MG, and CL: writing – reviewing and editing. All authors contributed to the article and approved the submitted version.

## Conflict of Interest

The authors declare that the research was conducted in the absence of any commercial or financial relationships that could be construed as a potential conflict of interest.

## Footnotes

1 http://www.genome-engineering.org/crispr/

2 https://www.ncbi.nlm.nih.gov/assembly/GCF_002204375.1

3 http://arep.med.harvard.edu/CasFinder/
