# Supplementary material for: Generation of Gene-Knockout Mongolian Gerbils via CRISPR/Cas9 System
Source: Front Bioeng Biotechnol. 2020 Jul 8;8:780. doi: 10.3389/fbioe.2020.00780 (PMC7360674; doi:10.3389/fbioe.2020.00780)
Supplement: Supplementary file 1 [file Data_Sheet_1.DOCX]

**Generation of Gene-knockout Mongolian Gerbils via CRISPR/Cas9 System**

Yan Wang, Peikun Zhao, Zidai Song, Xiaoyan Du, Xueyun Huo, Jing Lu, Xin Liu, Jianyi Lv, Changlong Li^*^, Meng Guo^*^, Zhenwen Chen^#^

**Table S1. The DNA oligos for constructing sgRNA expressing vectors.**

| Oligo name | Sequence (5’ →3’ ) |
| --- | --- |
| *Cst3*-pX330-E1-1-F | CACCGCGCCATGAGCGAGTACAACA |
| *Cst3*-pX330-E1-1-R | AAACCTTGTTGTACTCGCTCATGGCGC |
| *Cst3*-pX330-E1-2-F | CACCGCCACAGCCGTGCCATACAGG |
| *Cst3*-pX330-E1-2-R | AAACCCTGTATGGCACGGCTGTGGC |
| *Apoa2*-pX330-E2-1-F | CACCGTTTCAGACCATGGCTGACTA |
| *Apoa2*-pX330-E2-1-R | AAACTAGTCAGCCATGGTCTGAAAC |
| *Apoa2*-pX330-E2-2-F | CACCGACCATGGCTGACTATGGCA |
| *Apoa2*-pX330-E2-2-R | AAACTGCCATAGTCAGCCATGGTC |

**Table S2. The PCR primers for sgRNA *in vitro* transcription.**

| Name | Sequence (5’ →3’ ) |
| --- | --- |
| *Cst3*-3at-E1-1-F | tTAATACGACTCACTATAGGgCGCCATGAGCGAGTACAACA |
| C*st3*-3at-E1-1-R | AAAAGCACCGACTCGGTGC |
| C*st3*-3at-E1-2-F | tTAATACGACTCACTATAGGgCCACAGCCGTGCCATACAGG |
| C*st3*-3at-E1-2-R | AAAAGCACCGACTCGGTGC |
| *Apoa2*-3at-E2-1-F | tTAATACGACTCACTATAGGgTTTCAGACCATGGCTGACTA |
| *Apoa2*-3at-E2-1-R | AAAAGCACCGACTCGGTGC |
| *Apoa2*-3at-E2-2-F | tTAATACGACTCACTATAGGGACCATGGCTGACTATGGCA |
| *Apoa2*-3at-E2-2-R | AAAAGCACCGACTCGGTGC |

**Table S3. The primer sequences for genotype identification.**

| Name | Sequence (5’ →3’) |
| --- | --- |
| *Cst*3-GI-F | ATGGCTAGCCCACTACGATCC |
| *Cst*3-GI-R | TCTGCTTACGTGCCTTCACC |
| *Apoa2*-GI-F | GGGTGAGTCCTGTGGGATGT |
| *Apoa2*-GI-R | TTATCCTCTCAGCAGTCCCGT |

**Table S4. The summary of predicted off-target sites screened by using the algorithms referring to CasFinder.**

| Gene | The number of mismatch base pairs | | | | | Total |
| --- | --- | --- | --- | --- | --- | --- |
|  | 0 | 1 | 2 | 3 | 4 |  |
| *Cst3* | 0 | 0 | 1 | 6 | 32 | 39 |
| *Apoa2* | 0 | 0 | 2 | 10 | 71 | 83 |
| Total | 0 | 0 | 3 | 16 | 103 | 122 |

**Table S5. The primer sequences for amplifying** **the Cas9/*Cst3* sgRNA-mediated potential off-target sites*.***

| Name | Forward primer (5’ →3’) | Reverse primer (5’ →3’) |
| --- | --- | --- |
| OT-C1 | TGGCGCACATAAATGCACTC | CGGTACTGGGAGGAAACACG |
| OT-C2 | GGTCTTCAGCCTTCGGTCTC | CAGGCCTCTTTGCAGTGGAT |
| OT-C3 | GCGCTCTTTGCTCTGGTATG | ATTGGACGGCTGCTTCTTTG |
| OT-C4 | CAGGGGACTCCTGTCACTCT | ACTACGGCACAAGCAGGAAT |
| OT-C5 | AAATTCCTCGAGTCAGGGTCT | CTCCTGCCTCCTTCTAGGTTC |
| OT-C6 | CCCTAGTCACCAAACACCCA | TGGCTCTCCATCACGAATGT |
| OT-C7 | GCGGGGAGAACCATGCATTA | TCGCAGTTCTCTAAAGGGGC |
| OT-C8 | GAGGGCAGCAAACATTCCATT | GTGGGAAGGAGCAGGAAAGAAT |
| OT-C9  OT-C10 | AGTGCACAAACGATACTGATGC  TGGGTAGCTGATCCACTCACT | TCGAAGCTACCCAGGAATGC  ACACTGTGTCAAGGGACATCAA |

**Table S6. The primer sequences for amplifying the Cas9/*Apoa2* sgRNA-mediated potential off-target sites*.***

| Name | Forward primer (5’ →3’) | Reverse primer (5’ →3’) |
| --- | --- | --- |
| OT-A1 | TCGTGTTATTCAGCCTAATCTGT | ACACCGCTGCTTACTTCTGT |
| OT-A2 | TCACGCTCTGATTGGTCTGT | TAAAGACCCTGACCCTGGGA |
| OT-A3 | ATACCTCCAGACTGCACAGC | ACTCTCGTGTCATTGAGGACT |
| OT-A4 | ACCTCCACAATAAACATAGGCT | GATCGGCATGTGCTACTGGA |
| OT-A5 | GCCTTTGCTGGTCTGAGTTTG | AACAGGAAGGTAAGCAGCACAA |
| OT-A6 | GTTGCACTGTCCGTTTCCAG | GTGGTCCAATGAGCCCCATC |
| OT-A7 | CCATGCTGACCTTGAATTTAGAA | CCTAGCCCCCACCACCTA |
| OT-A9  OT-A10 | CAGCACTTGAGAACCGGGA  CATTAAAAGCACGGGCCACC | AGACCCTGCTCACCCTTCAA  CCACCTGGTTAGGAAGCCTG |

Fig. S1


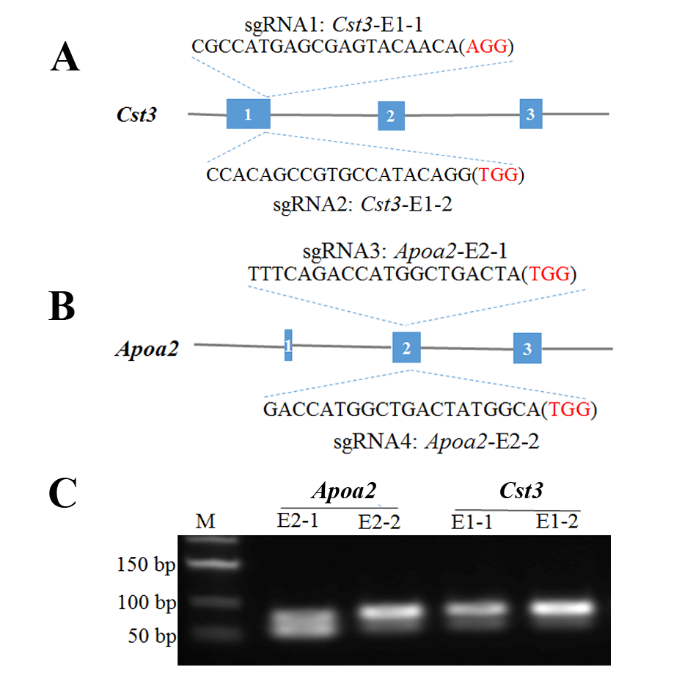


**Figure S1. RNA electrophoresis was employed to assess the length and integrity of the sgRNAs obtained by *in vitro* transcription.**

Fig. S2


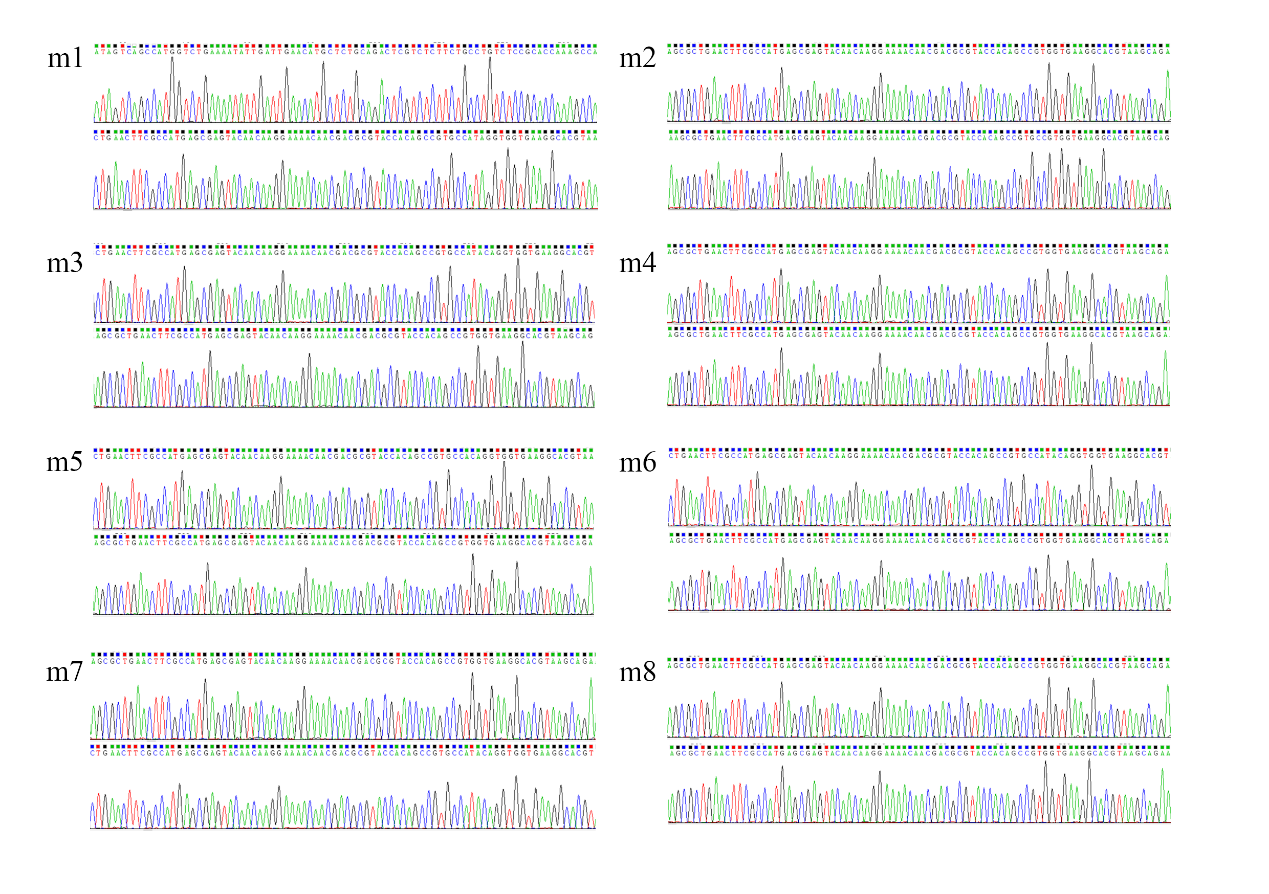


**Figure S2.** **Clone sequencing analysis of** **all *Cst3*-KO gerbil founders.**

Fig. S3


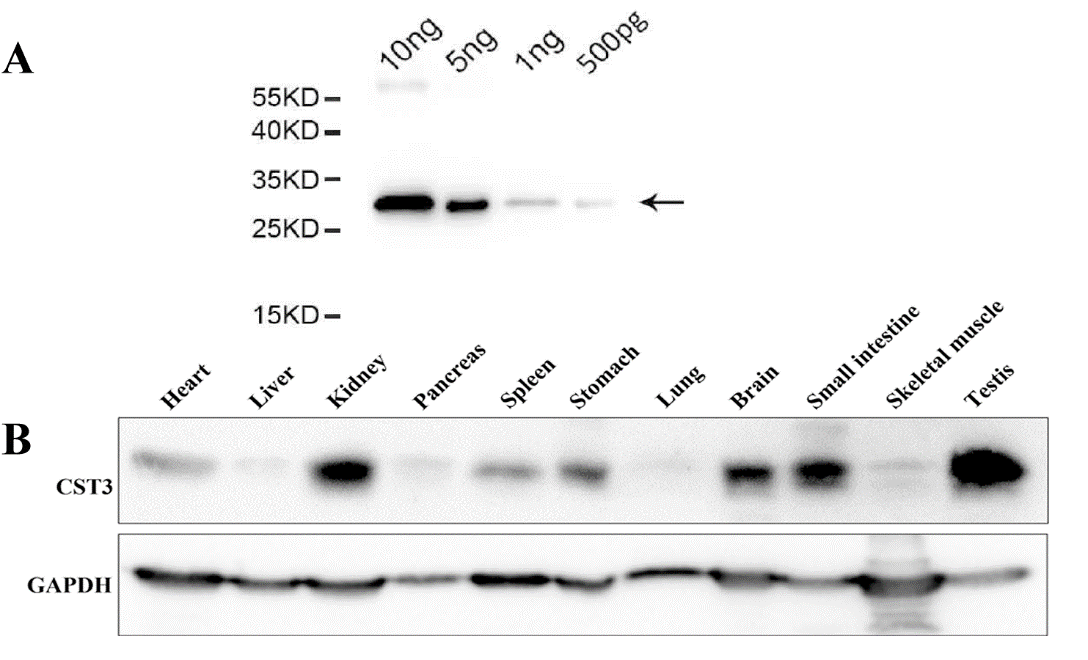


**Figure S3. The specificity of the anti-Mongolian gerbil CST3 antibody.** Western blotting of 10 ng, 5 ng, 1 ng, and 500 pg of CST3 antigen by using the rabbit anti-Mongolian gerbil CST3 polyclonal antibody (dilutions: 1:1000) (A). Western blotting of CST3 protein in various gerbil tissues by using the rabbit anti-Mongolian gerbil CST3 polyclonal antibody (dilutions: 1:1000) (B).

Fig. S4


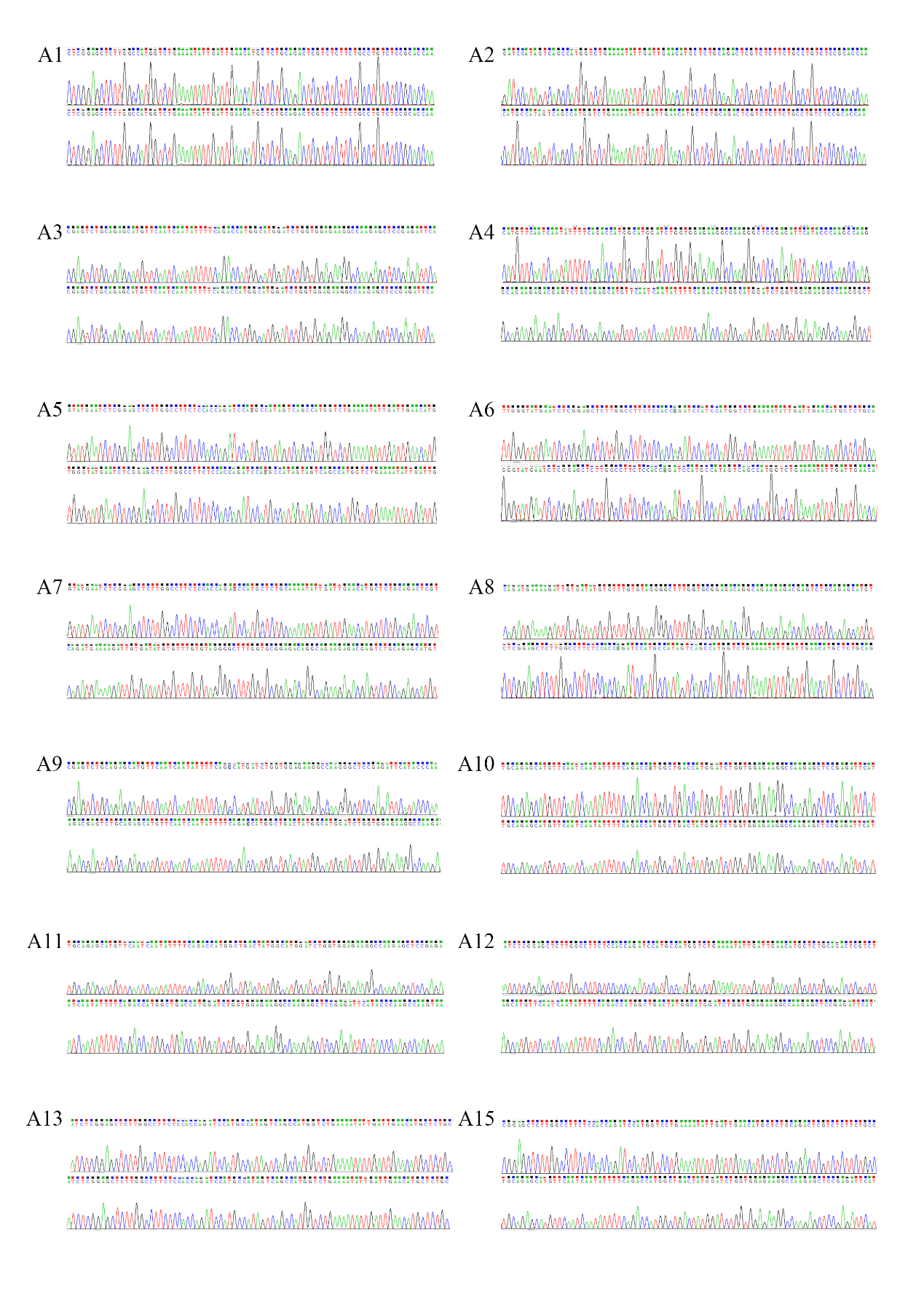


**Figure S4. Clone sequencing analysis of all** ***Apoa2*-KO gerbil founders.**

Fig. S5

**

 Figure S5. The potential off-target sequence prediction and off-target analysis in *Cst3*-KO founders.** The sequences of potential off-target sites of *Cst3* were predicted by CasFinder (A). Red lowercase letters were mismatched sites. PCR amplification and agarose electrophoresis (B), and Sanger sequencing (C) of the potential *Cst3* off-target sites were carried out.

Fig. S6


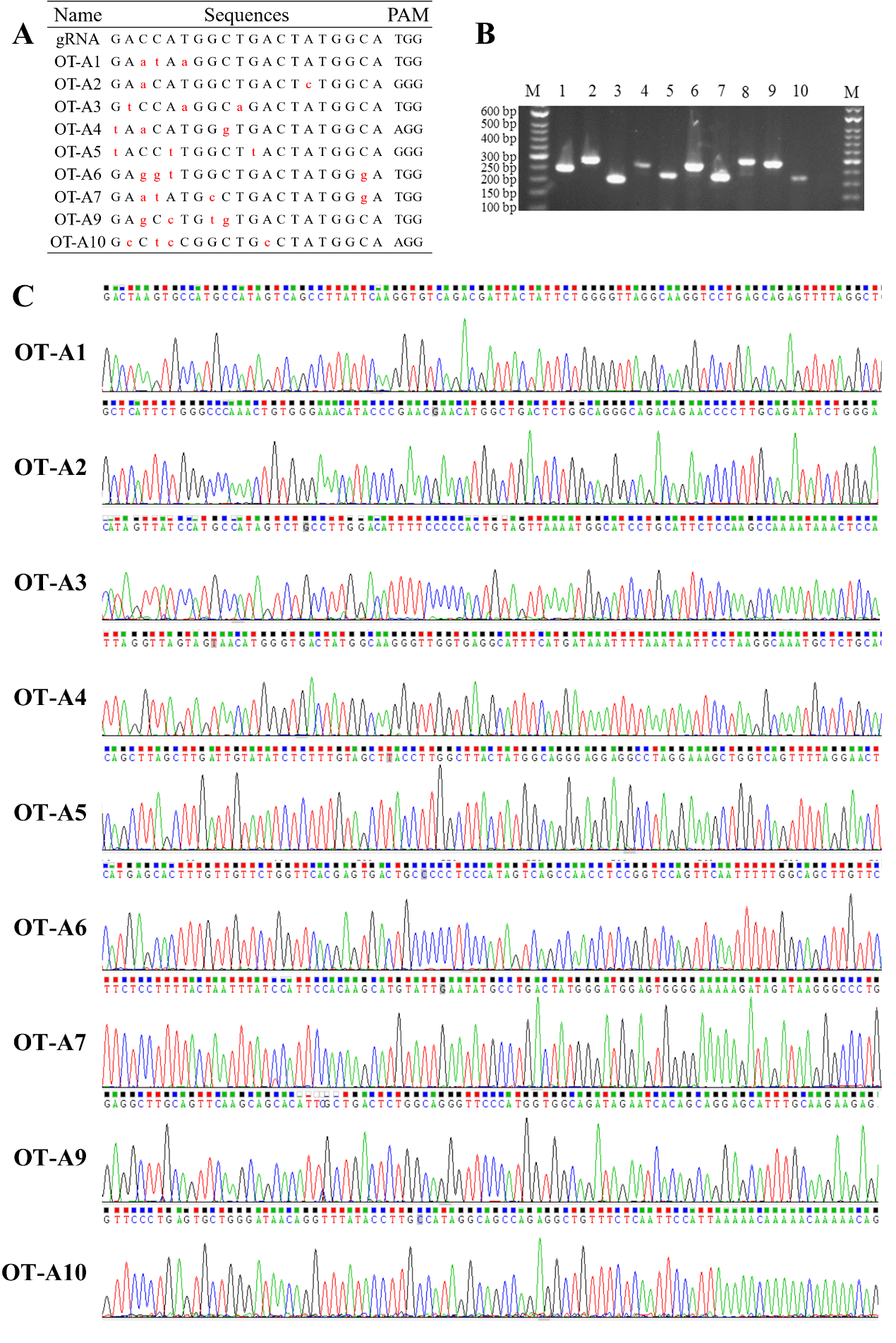
 **Figure S6. The potential off-target sequence prediction and off-target analysis in *Apoa2*-KO founders.** The sequences of potential off-target sites of *Apoa2* were predicted (A). Red lowercase letters were mismatched sites. PCR amplification and agarose electrophoresis (B), and Sanger sequencing (C) of the potential *Apoa2* off-target sites were carried out.

Fig. S7


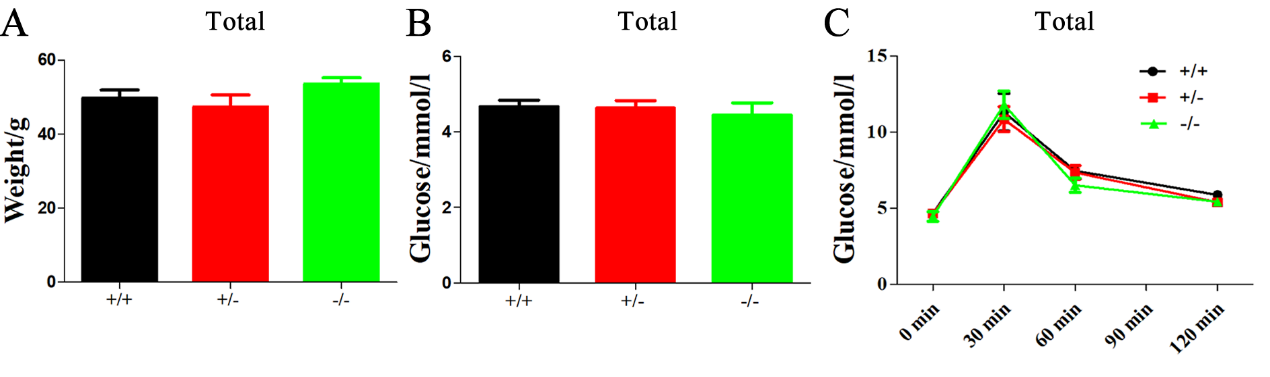


**Figure S7. Body weight, blood glucose and oral glucose tolerance test (OGTT) in *Apoa2-*KO gerbils.** Body weight (A), fasting blood glucose levels (B), and OGTT (C) of *Apoa2-*KO gerbils were measured, respectively. n=6.
